# Role of Hypoxia-Associated Long Noncoding RNAs in Cancer Chemo-Therapy Resistance

**DOI:** 10.3390/ijms26030936

**Published:** 2025-01-23

**Authors:** Muhammad Affan Elahi, Aamira Tariq, Ambrin Malik, Mahmoud Zhra

**Affiliations:** 1 Department of Biochemistry and Molecular Medicine, College of Medicine, Alfaisal University, Riyadh 11533, Saudi Arabia; melahi@alfaisal.edu; 2 Department of Biosciences, COMSATS University Islamabad, Islamabad Campus, Islamabad 45550, Pakistan; ambrin737@gmail.com; 3 Department of Anatomy and Genetics, College of Medicine, Alfaisal University, Riyadh 11533, Saudi Arabia; mzahra@alfaisal.edu

**Keywords:** hypoxia, HIF1-α, epithelial-to-mesenchymal transition (EMT), long noncoding RNA (LncRNA), chemotherapy resistance

## Abstract

Hypoxia is a well-known characteristic of the tumor microenvironment which significantly influences cancer development and is closely linked to unfavorable outcomes. Long noncoding RNAs (lncRNAs), which are part of the noncoding genome, have garnered increasing attention because of their varied functions in tumor metastasis. Long noncoding RNAs (lncRNAs) are defined as noncoding RNAs which are longer than 200 nucleotides, and they regulate diverse cellular processes by modulating gene expression at the transcriptional, post-transcriptional and epigenetic levels. Hypoxia is a well-established environmental factor which enhances the metastasis of solid tumors. Epithelial-mesenchymal transition (EMT) represents one of the key mechanisms triggered by hypoxia which contributes to metastasis. Numerous lncRNAs have been identified as being upregulated by hypoxia. These lncRNAs significantly contribute toward cancer cell migration, invasion and metastasis. Recent studies have identified a crucial role for these hypoxia-induced lncRNAs in chemotherapy resistance. These hypoxia-related lncRNAs can be plausible therapeutic targets for devising effective cancer therapies.

## 1. Introduction

Tumor cells grow in a dynamic and complex microenvironment termed the tumor microenvironment (TME). The TME comprises cytokines, hormones, enzymes, growth factors, diverse cell types like fibroblasts, immune inflammatory cells, pericytes and endothelial cells [1,2]. Hypoxia is referred to as a condition of insufficient oxygen when the tumor’s vascular supply is interrupted or the tumor outgrows its vascular supply [3]. Tumor cells farther than 180 μm from the vessel die by necrosis [4], whereas the cells in the immediate vicinity tend to survive in a chronic hypoxic condition eliciting a strong cellular hypoxic response [5]. Hypoxia leads to metabolic reprogramming, including a shift from aerobic to anaerobic respiration, pH regulation, growth factor production and cell proliferation, which are classical features of the tumor microenvironment which profoundly impact gene expression, leading to greater phenotypic heterogeneity, tumor progression and therapeutic response [6,7]. Different transcriptional and epigenetic mechanisms regulate hypoxia-induced gene expression and tumor metastasis. Cells respond to hypoxia via activation of the hypoxia signaling pathway (HSP). This cellular response to hypoxia is governed by a family of transcriptional factors called hypoxia-inducible factors (HIFs) [8,9]. Long noncoding RNAs (lncRNAs) have emerged as important regulators, influencing cancer cell migration and metastasis through mechanisms such as modulation of epithelial-mesenchymal transition (EMT) [10]. Notable lncRNAs like ANRIL, HOTAIR, MALAT1 and NEAT1 are upregulated by hypoxia and play crucial roles in tumor behavior [11,12,13,14]. Chemotherapy resistance continues to challenge the treatment of various cancers, including breast cancer, lung cancer, liver cancer and chronic lymphocytic leukemia, ultimately leading to poorer patient outcomes and higher mortality rates [15]. Recent studies indicated that hypoxia-induced lncRNAs are key mediators of this resistance, highlighting their potential as therapeutic targets [16,17]. Understanding the interplay between hypoxia, lncRNAs and chemotherapy resistance is essential for developing effective treatment strategies. This review highlights the role of hypoxia-associated lncRNAs in chemotherapy resistance while also exploring their diverse functions in cancer progression and metastasis.

## 2. HIF Subunits and Composition

**Figure 1:**
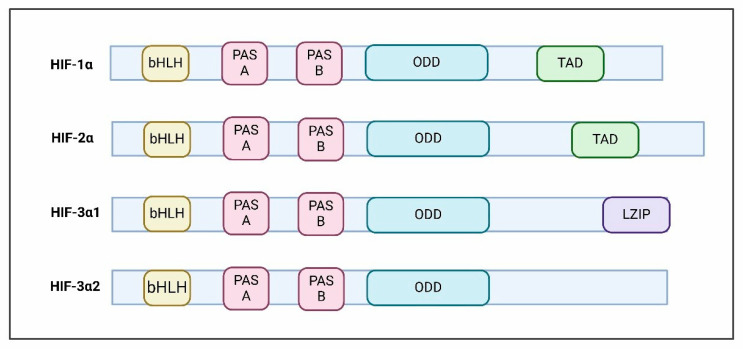
The composition of HIF subunits varies. The N-terminal structure is conserved among different HIF proteins, whereas the C terminal is different in different HIF isoforms. The structures of various HIF isoforms are illustrated. The basic helix–loop–helix (bHLH) and Per-ARNT-Sim (PAS) domains are crucial for both DNA binding and dimerization. Within HIF-α subunits, the oxygen-dependent degradation domain (ODD) provides sensitivity to oxygen levels. Importantly, the ODD region consists of conserved proline residues which are hydroxylated in an oxygen-dependent manner by prolyl hydroxylase (PHD) enzymes. Furthermore, the transactivation domain (TAD) plays a vital role in achieving maximum transcriptional activity. HIF-3α exhibits greater complexity due to its multiple splice variants, which have varying biological effects. Significantly, HIF-3 is distinct among the subunits as it contains a unique LZIP motif which also allows for DNA binding.

Hypoxia-inducible factor (HIF) is a heterodimer comprising basic helix loop helix and PERN-ARNT-SIM (bHLH-PAS) family proteins, comprising an inducible, oxygen–regulated α subunit and a stable, constitutive β subunit [18]. In humans, there are three α (HIF1-α,HIF2-α and HIF3-α) and two beta (ARNT and ARNT2) paralogs [19]. HIF1-α was initially identified by the expression of the erythropoietin gene [20]. Structurally, all HIF alpha subunits share common functional domains (Figure 1). The N-terminal domain comprising bHLH serves as a DNA-binding domain [21], whereas the PAS domain harboring two vital regions, PAS-A and PAS-B, plays a crucial role in its dimerization [19] (Figure 1).

The alpha subunit bears an oxygen-dependent degradation domain (ODD) rendering HIF1-α sensitivity toward oxygen [22], whereas the C-terminal transcriptional activation domain (TAD) is crucial for full transcriptional activation [23]. Meanwhile, HIF1-α and HIF2-α are master regulators of hypoxic responses which bear a significant degree of sequence similarity and share common target genes. However, they exhibit distinct DNA binding patterns and also modulate distinct gene targets [24]. HIF1-α exhibits ubiquitous expression, whereas HIF2-α demonstrates a tissue-specific pattern and varied activation kinetics [25]. Both isoforms are overexpressed in different cancers with unfavorable prognoses and associated with chemo- and radiotherapy resistance [26]. Furthermore, HIF1-α enhances glycolytic gene expression, facilitating anaerobic metabolism. Moreover, HIF1-α regulates angiogenesis, which is vital for cancer cell survival and growth in low-oxygen environments [27,28]. On the other hand, HIF2-α promotes cell proliferation and is crucial in tumorigenesis and metastasis, thus influencing cancer progression and severity [29,30].

HIF3-α is the least explored and tissue-specific factor, demonstrating complex regulation due to the generation of multiple variants stemming from different promoters, transcription initiation sites, and alternative splicing [31]. Some of these variants lack oxygen sensitivity and a DNA-binding ability [32]. Recent studies have challenged the prior assumption that HIF3-α primarily suppresses the expression of genes regulated by HIF-1α and HIF-2α. Instead, HIF3-α has been shown to act as an oxygen-sensitive transcriptional activator, managing a unique array of target genes in response to low oxygen levels and influencing tumor immune evasion [32,33]. Furthermore, HIF-3α exhibits tissue-specific expression and is modulated during development, and its dysregulation has been implicated in multiple cancer types [34]. Notably, HIF3-α has also been implicated in promoting pancreatic cancer metastasis through transcriptional activation of the RhoC-ROCK1 pathway [35]. HIFs are vital in numerous physiological and pathological situations, such as congenital defects, ongoing inflammation and cancer, which positions them as promising targets for therapeutic interventions.

## 3. Oxygen-Dependent Regulation of HIFs

**Figure 2:**
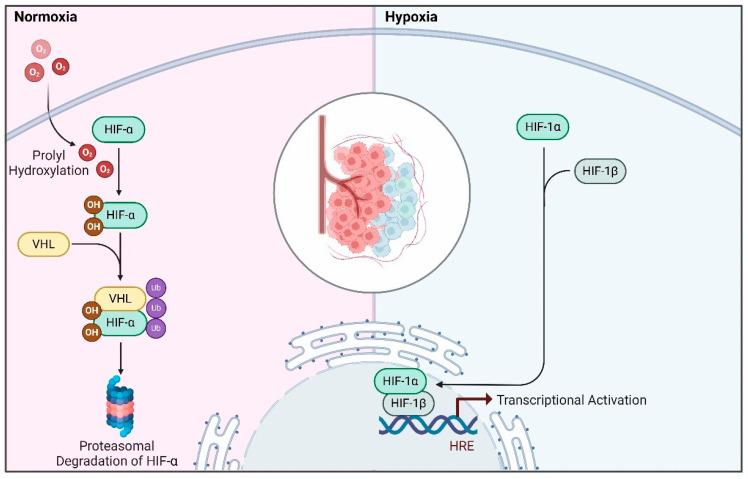
**Regulation of HIF through oxygen levels.** In normal oxygen situations, prolyl hydroxylase (PHD) enzymes hydroxylate HIF-α subunits in a process which depends on oxygen. This hydroxylation tags them for degradation via the proteasome by increasing their affinity for the E3 ligase VHL. Conversely, in low-oxygen conditions, PHD activity is suppressed, resulting in stabilization and movement of HIF-α subunits into the nucleus. Once there, they form a heterodimer with HIF-1β and initiate the transcription of genes which facilitate adaptation to hypoxic environments. HIF refers to hypoxia-inducible factor, and VHL stands for the von Hippel-Lindau protein.

Under normoxic conditions, the HIF1-α subunits are quickly hydroxylated by a group of dioxygenases known as prolyl hydroxylase domain-containing enzymes (PHD1, 2 and 3 or EGLN1–3), which belong to the Egl-9 family [36]. These hydroxylated HIF1-α subunits are then recognized by the von Hippel-Lindau (VHL) tumor suppressor protein and E3 ubiquitin ligase complex, leading to the rapid degradation of HIF1-α via the proteasome pathway (Figure 2) [7]. When hypoxia occurs, the activity of PHD enzymes is inhibited. This inhibition causes the stabilization and accumulation of HIF1-α in the nucleus, where it forms a stable complex with the β subunit. This HIF complex binds to specific DNA sequences containing the consensus nucleotide sequence 5′-(A/G)CGTG-3′, located within the hypoxia response elements (HREs) in the promoter regions of HIF1 target genes, facilitating the stimulation of downstream transcription (Figure 2) [37,38]. Notably, in mammalian systems, it has been demonstrated that HIF1 can function as either an activator or repressor, depending on the cellular context, and the set of genes regulated by HIF1 varies among different cell types [39]. Overall, hypoxia induces a finely tuned HIF-dependent transcriptional activation which triggers a wide range of cellular adaptations, including metabolic reprogramming, increased proliferation, reduced apoptosis, angiogenic growth and the promotion of cell survival under these conditions [40,41].

Aside from post-transcriptional regulation, the expression of HIF is also delicately adjusted at the translational stage through a complex interaction with the mammalian target of rapamycin (mTOR) complex [42], where mTOR is a serine/threonine kinase which acts as a sensor and integrator of signals from the surrounding extracellular environment. It comprises two functionally distinct complexes: mTOR complex 1 (mTORC1) and mTOR complex 2 (mTORC2) [43]. While mTORC1 mainly controls cell growth and metabolism by affecting protein translation, mTORC2 is involved in regulating cell survival and proliferation [44]. In various cancer types, the mTOR complex is often over-activated due to the loss of tumor suppressors like phosphatase and tensin homologue (PTEN), activation of oncogenes such as phosphoinositide 3-kinase (PI3K) and metabolic reprogramming [45]. Numerous studies have highlighted the crucial role of mTORC1 in modulating HIF1-α protein expression at both the translational and transcriptional levels [46]. Conversely, hypoxia and the presence of HIF1-α negatively regulate mTOR activity by inducing transcription of the mTOR inhibitor REDD1, which leads to a halt in translation [47]. Interestingly, the activation of HIF2 has the opposite effect, as the stabilization of HIF2 enhances mTORC activity by regulating the amino acid transporter SLC7A5 through transcription, resulting in increased amino acid uptake, the activation of mTORC1 and enhanced tumor growth in xenograft models [48].

The activation of HIF1 pathways is correlated with a more aggressive tumor phenotype and unfavorable clinical outcomes across various cancer types. Thus, investigating the regulatory mechanisms which govern HIF1-mediated transcriptional control could enhance our understanding of the role of hypoxia in tumor progression. The genes produced as a result not only influence various biological processes, such as cellular metabolism, growth, apoptosis, and migration, but also include several oncogenes and tumor suppressor genes. Given that these processes and genes play pivotal roles in carcinogenesis, it is not unexpected that hypoxia serves as a crucial factor in the tumor microenvironment associated with cancer development.

## 4. Hypoxia and Chemotherapy Resistance

Hypoxia-inducible factors (HIFs) function as sensors for low oxygen levels, coordinating a collective response which enhances the survival and invasiveness of cancer cells while also influencing a wide range of metabolic adaptations. This results in promoting tumor growth and resistance to chemotherapy. The rise in the uptake of glucose and amino acids, the flow of glycolysis and the production of lactate; the changes in glutamine metabolism, the tricarboxylic acid cycle and oxidative phosphorylation; the elevated levels of reactive oxygen species in mitochondria; and the adjustments in both fatty acid synthesis and breakdown are characteristic features of the metabolic reconfiguration caused by hypoxia.

In addition to factors independent of metabolism, one fundamental reason for the ineffectiveness of chemotherapy is the structure of tumors. Specifically, in solid tumors, cells located in hypoxic regions, often distant from blood vessels, experience restricted delivery of chemotherapeutic agents due to irregular vascularization [49]. Furthermore, HIF1-α enhances the expression of multiple genes which contribute to resistance against chemotherapy. One such gene is MDR1, which encodes P-glycoprotein (Pgp) [50]. Pgp is a member of the adenosine triphosphate (ATP)-binding cassette (ABC) transporter family and acts to efflux various chemotherapeutic drugs regardless of their structure and mechanism of action, thus resulting in a multidrug resistance (MDR) phenotype [51]. The upregulation of Pgp driven by HIF1-α has been observed in several cancers, including liver [52], laryngeal, lung [53] and breast [54] cancers, along with malignant pleural mesothelioma [55] and chronic lymphocytic leukemia in B-cells [56]. The wide variety of tumors exhibiting this phenomenon indicates that it is a fairly conserved mechanism. Additionally, other ABC transporters involved in drug efflux, such as MDR-related protein 1 (MRP1) [57], lung resistance protein (LRP) [58] and breast cancer resistance protein (BCRP) [59], are also regulated at the transcriptional level by HIF1-α and HIF2-α, further increasing the range of drugs that are effluxed and thus lose effectiveness in hypoxic areas.

The changes in metabolism within glycolysis and the mitochondria triggered by low oxygen levels contribute to chemoresistance through different mechanisms like increased pro-survival mechanisms and diminished apoptosis, activation of epithelial-to-mesenchymal transition (EMT), heightened DNA repair capabilities, modifications in drug metabolism and alterations in drug targets [60,61]. Notably, hypoxia alters mitochondrial dynamics, including the fusion, fission and mitophagy processes, which are critical for cellular survival in low-oxygen environments. Mitochondrial dysfunction, characterized by diminished mitochondrial DNA and alterations in metabolic pathways, promotes EMT, thereby enhancing drug resistance and metastasis [58,62]. Furthermore, modifications in mitochondrial activities, such as apoptosis, ROS generation and calcium signaling pathways, are significant contributors to chemoresistance [63].

One significant contributor to chemoresistance is the heightened acidification of the tumor microenvironment, resulting from increased glycolytic enzyme activity and MCT4 expression [64]. The decreased extracellular pH (pHe) promotes the protonation of weak bases like anthracyclines, leading to their inactivation and entrapment in lysosomes after they have infiltrated the cancer cell. This process is referred to as “ion trapping” [65]. Furthermore, hypoxic and acidic tumor regions exhibit enhanced expression of enzymes which promote alkalinization. The Na^+^/H^+^ exchanger (NHE) serves as a prime example of a transporter which is upregulated in response to the surrounding acidity. Its activity leads to an increase in the intracellular pH (pHi), establishing optimal conditions for the efficient efflux of Pgp, which operates best at a pH level of 7.6–7.8 [66].

Moreover, chemoresistance in hypoxic tumor regions is associated with alterations in mitochondrial metabolism, characterized by reduced oxidative phosphorylation (OXPHOS) and heightened mitophagy, resulting in decreased ATP synthesis [67,68,69]. Despite the anticipation of decreased ATP for ABC transporters under such conditions, cellular metabolic adaptability promotes survival. The production of ROS, linked to OXPHOS changes in hypoxic tumor areas, may lead to mtDNA damage, thus impairing OXPHOS efficiency [70]. In general, the mitochondrial-related factors (reduced OXPHOS and ATP production, elevated ROS, heightened mitophagy and increased mtDNA mutations) which define hypoxic tumor cells contribute to chemoresistance. Cells which express HIF1-α display significant metabolic flexibility, enabling them to survive more effectively when faced with limited glucose and oxygen supplies or during chemotherapy treatments. Thus, the diverse impacts of hypoxia on metabolic reprogramming collectively play a role in chemoresistance through various but interrelated mechanisms.

## 5. Hypoxia-Associated Long Noncoding RNAs and Their Role in Gene Expression Regulation

The wide spectrum of hypoxia-responsive genomes is not limited to protein coding genes but also includes noncoding RNAs like microRNAs, lncRNAs and circular RNAs. Hypoxia responsive ncRNAs play a pivotal role in modulating hypoxic gene expression at the transcriptional and post-transcriptional levels, either by regulating the HIF transcriptional cascade or acting as HIF-guided effector molecules [71]. Recent studies have shown that long noncoding RNAs (lncRNAs) are crucial in gene expression regulation across diverse cellular processes, including migration, invasion, anti-apoptosis, angiogenesis and tumor progression [72].

The lncRNAs regulate gene expression or protein levels via different mechanisms. They may interact with the protein–protein complex, leading to its degradation or stability, dissociate protein binding to a promoter. The formation of cellular structures like paraspeckles triggers protein glycosylation, regulates RNA splicing or serves as a coactivator of gene expression [73]. For instance, the lncRNA NEAT1 is a hypoxia-inducible lncRNA critical for the formation of paraspeckles, which can sequester RNA transcripts and transcriptionally active proteins, thereby modulating the expression of proteins involved in tumor metastasis [74]. Additionally, lncRNA MALAT1 reduces the degradation of HIF1-α and HIF2-α by inhibiting the binding of VHL protein to these factors [75]. The lncRNA H19 is responsible for the nuclear localization of HIF1-α [76]. Another hypoxia-inducible lncRNA, LUCAT1, interacts with poly-pyrimidine tract-binding protein (PTBP1) to alter the alternative splicing of DNA damage genes [77]. These findings underscore the significant role of hypoxia-inducible lncRNAs in cancer biology and their potential implications for therapeutic strategies.

## 6. Role of Hypoxia-Associated Long Noncoding RNAs in Cancer Metastasis

### 6.1. Breast Cancer

**Figure 3:**
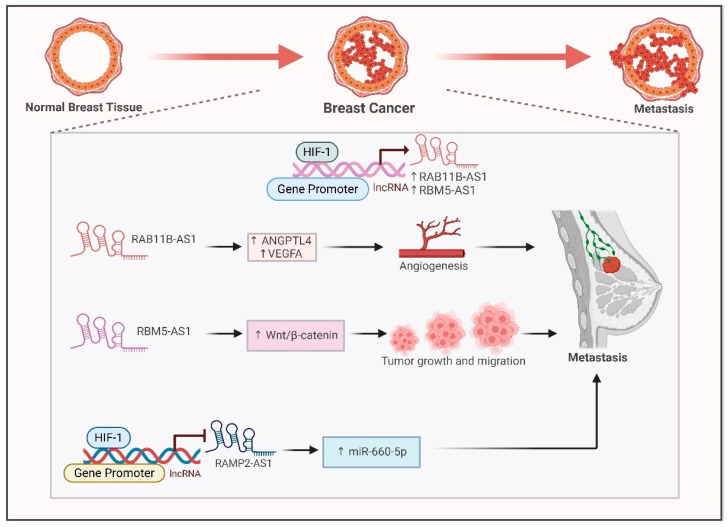
**Interaction between HIF1-α and hypoxia-associated lncRNAs in breast cancer (BC)**. HIF1-α directly binds to the promoters of RAB11B-AS1 and RBM5-AS1 to induce their expression. Both of these lncRNAs lead to breast cancer metastasis. HIF1-α suppresses the expression of tumor suppressor RAMP2-AS1 to upregulate oncogenic miR-660-5p and further promote BC metastasis.

Metastatic breast cancer spreads from the breast to different parts of the body, like the lungs, liver, bone and brain. Hypoxia is a hallmark of the breast tumor microenvironment, playing a pivotal role in cancer progression. Long noncoding RNAs (lncRNAs) are essential in the progression of breast cancer in low-oxygen environments, and lncRNAs which respond to hypoxia are linked to unfavorable prognoses and influence tumor biology, suggesting that they could be used as prognostic indicators and treatment targets. Hypoxia-associated lncRNAs contribute toward cancer metastasis through diverse mechanisms. Hypoxia induces the expression of RAB11B-AS1 in MDA-MB231 cells by binding HIF1-α to its promoter [78]. Overexpression of RAB11B-AS1 promotes invasion and migration. This promotion of angiogenesis and breast cancer metastasis is mediated by proangiogenic factors like ANGPTL4 and VEGFA [79]. Silencing of RAB11-B-AS1 promotes cancer cell apoptosis and reduces breast cancer metastasis (Figure 3) [79].

Hypoxia induces expression of the lncRNA RBM5-AS1 via the RUNX2 transcription factor [80]. RBM5-AS1 initiates Wnt/β-catenin signaling by upregulating and interacting with β-catenin. RBM5-AS1 downregulates the expression of AXIN1, leading to the accumulation of β-cateninin cytoplasm followed by its nuclear translocation [80]. RBM5-AS1 bears a binding site for β-catenin/TCF4 [80]. It acts as a scaffold and facilitates the organization of the β-catenin-TCF4 transcriptional complex. Thus, RBM5-AS1 plays a crucial role in the growth, maintenance of stemness, migration and invasion of breast cancer by enhancing the accumulation of β-catenin and strengthening the interaction between TCF4 and β-catenin, which activates the Wnt/β-catenin pathway (Figure 3) [80]. Contrary to hypoxia-induced lncRNAs, a recent study documented the hypoxia-suppressed lncRNA RAMP2-AS1. RAMP2-AS1 has been previously documented as a tumor suppressor in breast cancer by downregulating CXCL11 through DNMT1 and DNMT3b [81]. The knockdown of RAMP2-AS1 consequently upregulates miR-660-5p, leading to decreased expression of ATM in MCF-7 and MDA-MB231 cells. This lncRNA is downregulated in breast cancer compared with normal controls. It regulates the miR-660/ATM axis in breast cancer (Figure 3) [82]. These research findings underscore the intricate relationship between long noncoding RNAs (lncRNAs) and hypoxia in breast cancer, stressing their possible influence on prognoses, treatment strategies and patient outcomes. Additional studies could result in enhanced diagnostic, prognostic and therapeutic resources for individuals with breast cancer.

### 6.2. Hepatocellular Carcinoma (HCC)

**Figure 4:**
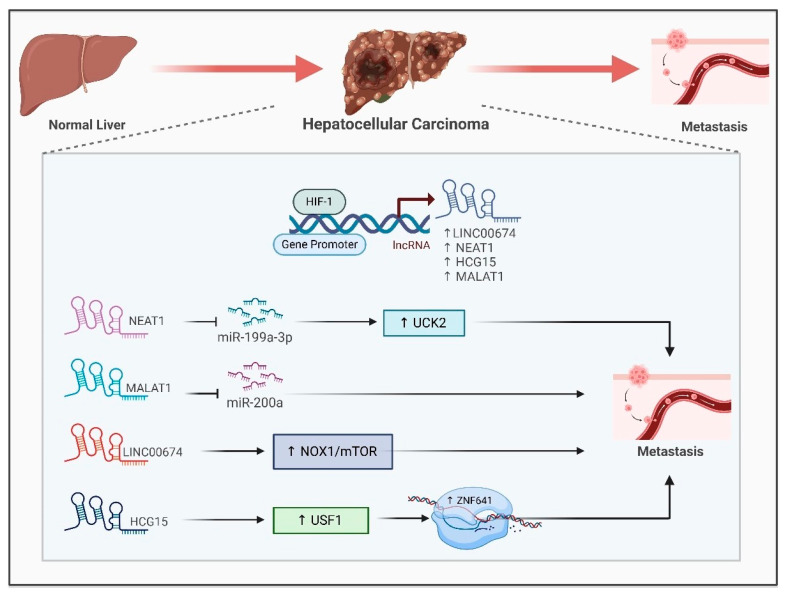
**Interaction between HIF1-α and hypoxia-associated lncRNAs in hepatocellular carcinoma (HCC)**. HIF1-α directly binds to the promoters of LINC00674, NEAT1, HCG15 and MALAT1 to induce their expression. NEAT1 and MALAT1 lncRNAs promote HCC metastasis by sponging miR-1991-3p and miR200a, respectively, leading to breast cancer metastasis. LINC00674 activates the NOX/mTOR pathway, and HCG15 enhances ZNF641 transcription to promote HCC metastasis.

Recent research has underscored the important function of hypoxia-associated long noncoding RNAs (lncRNAs) in hepatocellular carcinoma (HCC). These lncRNAs participate in multiple processes such as glucose metabolism, maintenance of cancer stem cells, cell apoptosis, proliferation and immune evasion, all of which contribute to unfavorable outcomes for HCC patients [83]. Scientists have discovered and confirmed lncRNA signatures associated with hypoxia as prognostic models for HCC, showing their ability to serve as independent predictors which surpass conventional clinical factors [84,85]. Hypoxia induces the expression of LINC00674 by increasing the occupancy of HIF1-α in the HRE region of its promoter. Overexpression of this lncRNA stimulates HCC cell proliferation and metastasis by activating NOX1/mTOR signaling (Figure 4) [86]. The lncRNA NEAT1 is another hypoxia-induced RNA which interacts with the tumor suppressive miR-199a-3p to sustain HCC growth by regulating the miR-199a-3p/UCK2 pathway (Figure 4), suggesting its potential to be a plausible therapeutic target [11]. The lncRNA MALAT1 regulates Hep3B cell proliferation, migration, invasion and metastasis by sponging miR-200a (Figure 4) [87]. Another hypoxia-responsive lncRNA, HLA complex group 15 (HCG15), facilitates HCC cell migration and invasion by enhancing ZNF641 transcription. The knockdown of HCG15 abolishes USF1-mediated ZNF641 transcription (Figure 4). leading to reduced Hep3B cell proliferation, migration and invasion [88]. These findings imply that lncRNAs associated with hypoxia may act as promising biomarkers and therapeutic targets for the diagnosis, prognosis and treatment of liver cancer.

### 6.3. Gastric Cancer (GC)

**Figure 5:**
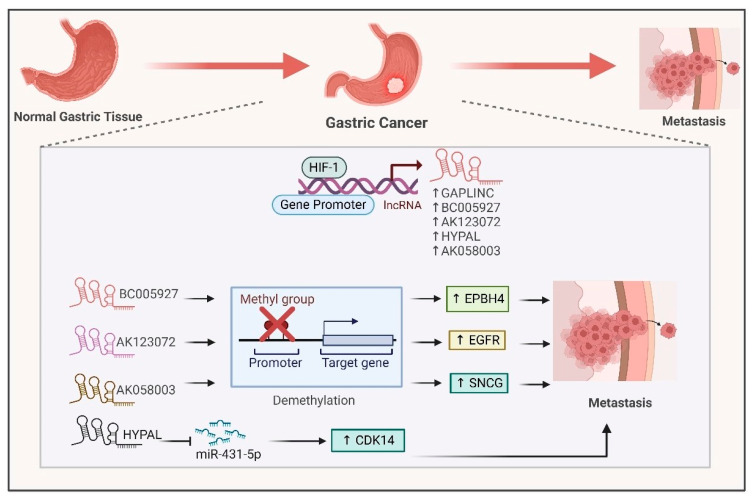
**Interaction between HIF1-α and hypoxia-associated lncRNAs in gastric cancer**. HIF1-α directly binds to the promoters of GAPLINC, BC005927, AK123072, HYPAL and AK058003 to induce their expression. The BC005927, AK123072 and AK0580031 lncRNAs boost the expression of oncogenic genes like EPBH4, EGFR and SNCG by demethylating their promoters. The lncRNA HYPAL acts as a ceRNA for miR-431-5p, resulting in overexpression of CDK14 and leading to metastasis.

Hypoxia plays a crucial role in gastric cancer progression by inducing the expression of different lncRNAs via HIF1-α-mediated transcriptional activation. For example, lncRNas like GAPLINC are overexpressed in gastric cancer, and hypoxia promotes its malignancy (Figure 5) [89]. The lncRNA BC005927 is a direct transcriptional target of HIF1-α. BC005927 upregulates the expression of metastasis-associated gene EPBH4 by modulating its DNA methylation, promoting gastric cancer metastasis (Figure 5) [90]. In another study, hypoxia induced expression of the lncRNA AK123072. High expression of this lncRNA promotes gastric cancer cell migration and invasion by modulating the expression of the metastasis-related gene EGFR (Figure 5). Depletion of AK123072 leads to increased methylation of the CpG island in the promoter region [91]. Another lncRNA, HYPAL, promotes GC metastasis under hypoxic conditions by sponging miR-431-5p, leading to upregulation of CDK14. This HIF1-α/HYPAL/miR-431-5p/CDK14 axis activates the Wnt-β-catenin pathway (Figure 5), promoting gastric cancer cell proliferation and inhibiting apoptosis [92]. In gastric cancer, hypoxia induces expression of the lncRNA AK058003. This lncRNA resides upstream of synuclein gamma (SNCG), a synuclein family member. It promotes the expression of SNCG via demethylation of its promoter (Figure 5), leading to an increase in cell proliferation, migration and invasion [93]. Thus, the lncRNAs which are regulated by hypoxia play various roles in metabolism, autophagy, invasion and metastasis within the hypoxic microenvironment of gastrointestinal cancers, positioning them as potential targets for therapy [94].

### 6.4. Pancreatic Cancer (PC)

**Figure 6:**
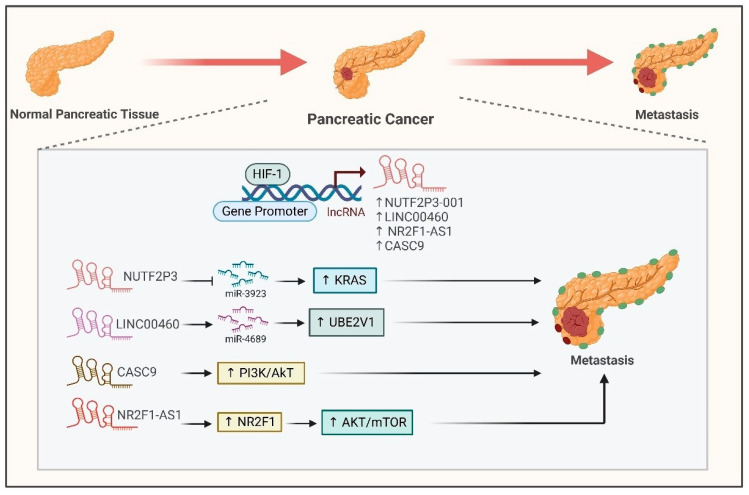
**Interaction between HIF1-α and hypoxia-associated lncRNAs in pancreatic cancer**. HIF1-α directly binds to the promoters of NUTF2P3-001, LINC00460, CASC9 and NR2F1-AS1 to induce their expression. NUTF2P3-001 and LINC00460 sponge miR-3923 and miR-4689 to enhance the expression of KRAS and UBE2V1, respectively, leading to pancreatic cancer metastasis. CASC9 and NR2F1-AS1 activate the PI3K/AKT and AKT/mTOR pathways, respectively, to promote pancreatic cancer metastasis.

Recent research has underscored the important function of long noncoding RNAs (lncRNAs) induced by hypoxia in the advancement of pancreatic cancer. Scientists have pinpointed various lncRNAs, such as NUTF2P3-001 [95], LINC00460 [96] and CASC9, which are elevated in hypoxic environments and play a role in tumor development (Figure 6). Hypoxia-inducible factor 1-α (HIF1-α) is crucial for the regulation of these lncRNAs as it attaches to their promoter regions. These lncRNAs facilitate the proliferation, invasion and metastasis of cancer cells via several mechanisms, including modulation of the miR-3923/KRAS pathway (Figure 6) [95], regulation of the miR-4689/UBE2V1 axis [96] and the enhancement of glycolysis and epithelial-mesenchymal transition (EMT) via activation of the PI3k/AKT pathway (Figure 6) [97].

In hypoxia, HIF1-α also induces the expression of NR2F1-AS1, which positively regulates its neighboring gene NR2F1, to promote cancer cell proliferation, migration and invasion by activating AKT/mTOR signaling (Figure 6) [98]. This evidence indicates that lncRNAs associated with hypoxia may act as potential therapeutic targets and prognostic markers for pancreatic cancer.

### 6.5. Lung Cancer

**Figure 7:**
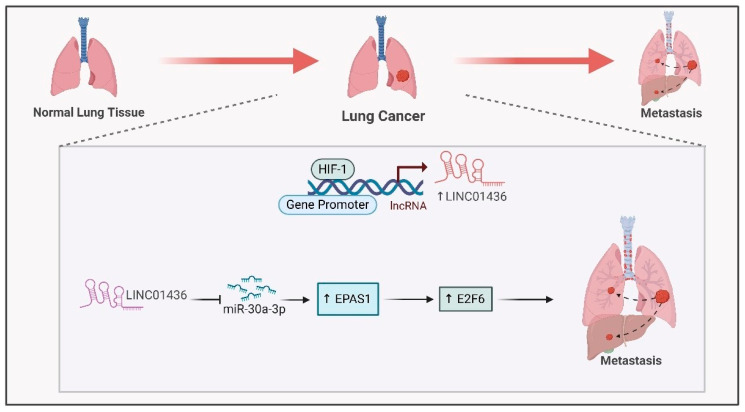
**Interaction between HIF1-α and hypoxia-associated lncRNAs in lung cancer**. Hypoxia induces the expression of LINC01436 through the binding of HIF1-α to its promoter. LINC01436 sponges miR-30a-3p to activate E2F6, leading to lung cancer metastasis.

Long noncoding RNAs (lncRNAs) are essential in driving cancer progression under hypoxic conditions, especially in lung cancer. Hypoxia-responsive lncRNAs (HRLs) influence gene expression through various mechanisms and affect HIF1 signaling [7]. In non-small cell lung cancer (NSCLC), LINC01436 functions as an oncogene by sequestering miR-30a-3p and is regulated by E2F6 in response to hypoxia [99]. LINC01436 sponges miR-30a-3p to regulate the expression of its target gene EPAS1 (Figure 7) [99], promoting lung cancer cell migration and invasion and tumor metastasis [99].

A set of seven hypoxia-related lncRNAs have been identified as prognostic indicators for patients with lung adenocarcinoma, showing a connection to immunosuppressive environments [100]. The relationship between lncRNAs and hypoxia affects numerous facets of tumor development, including growth, metabolism, angiogenesis and metastasis [101]. These results underscore the potential of hypoxia-related lncRNAs as biomarkers and therapeutic targets in lung cancer, paving the way for innovative diagnostic and treatment strategies.

In general, hypoxia-induced lncRNAs share several common mechanisms which facilitate cellular invasion and proliferation and promote chemoresistance across a spectrum of cancer types [102]. These lncRNAs regulate gene expression at multiple levels, including the epigenetic, transcriptional and post-transcriptional levels, by interacting with DNA, RNA and proteins [103]. Evidence suggests that these lncRNAs, either through direct interaction or by modulating specific intermediate miRNAs, enhance the expression of various factors associated with angiogenesis, cellular proliferation and differentiation. Additionally, they exert influence over drug transporters and multidrug resistance proteins while also upregulating cancer-associated signaling pathways, including mTOR and Wnt/β-catenin [104].

## 7. Role of Hypoxia-Induced Long Noncoding RNAs in Chemoresistance

The presence of low oxygen levels in the tumor microenvironment plays a role in the development of resistance to chemotherapy across different types of cancer. Numerous studies have pointed out that long noncoding RNAs (lncRNAs) are significant factors in this phenomenon. Profiling methods and bioinformatics analysis have enabled us to discover an increasing number of hypoxia-regulated noncoding RNAs by identifying the presence of hypoxia response elements (HREs) in their promoter regions [37]. Additionally, various studies have shown that certain noncoding RNAs can be induced by hypoxia even in the absence of HREs, suggesting an indirect regulatory mechanism which often involves epigenetic factors. Hypoxia-inducible factor (HIF) may influence the expression of noncoding RNAs by activating histone deacetylases or impacting the machinery responsible for microRNA (miRNA) maturation [105]. Research indicates that lncRNAs induced by hypoxia play a role in chemoresistance through multiple mechanisms, such as modulation of drug efflux proteins, promotion of epithelial-mesenchymal transition (EMT), and sponging of miRNAs, highlighting possible therapeutic targets to address chemoresistance in tumors affected by hypoxia. In this review, some of the hypoxia-induced lncRNAs were associated with chemoresistance.

### 7.1. Long Noncoding RNA H19

The long noncoding RNA H19 plays a significant role in chemoresistance across various cancers, including glioma, breast cancer and colorectal cancer. H19 expression is upregulated in chemoresistant cells and tumors [106]. Different studies indicated that H19 knockdown enhances sensitivity to various chemotherapeutic agents, including temozolomide, doxorubicin and paclitaxel [106,107,108,109]. In non-small lung cancer (NSCLC) cell lines (A549 and H1650), hypoxia induces the expression of lncRNAs H19, HIF1-α, HO-1, MRP-1 and P-gp, resulting in increased migration and invasion of cancer cells along with an increase in cisplatin resistance [107].

According to recent studies, MRP, a multidrug-resistant membrane transporter protein, is closely related to tumor multidrug resistance. MRP1 overexpression is one of the primary mechanisms which causes multidrug resistance in lung cancer cells. HO-1 overexpression promotes invasion and metastasis in lung cancer. P-glycoprotein (P-gp), when overexpressed, increases drug efflux by enhancing the action of the efflux pump. This reduces the accumulation of drugs in cells and diminishes the effectiveness of chemotherapy on tumor cells. In NSCLC cell lines (A549 and H1650), the expression of lncRNAs H19, HIF1-α, HO-1, MRP-1, and P-gp was induced under hypoxic conditions, compared with normoxic conditions. Furthermore, hypoxia promoted migration and invasion in NSCLC cells, while the apoptosis rate of cancer cells exposed to cisplatin decreased [110]. In another study, increased expression of H19 was reported in colorectal cancer (CRC) under hypoxic conditions or in oxiplatin-treated CRC cells. H19 plays a pivotal role in oxaliplatin resistance in CRC both in vitro and in vivo. H19 acts as a competitive endogenous lncRNA (celncRNA) of miR-675-3p to regulate EMT in CRC [17]. Experiments conducted on CRC cell lines HCT116 and HT29 suggest that H19 knockdown enhances oxaliplatin efficacy and partially blocks EMT. H19 knockdown in a xenograft tumor model promotes the efficacy of oxaliplatin treatment. Meanwhile, miR-675-3p mimics reduce oxaliplatin toxicity, increase vimentin protein expression and decrease E-cadherin expression. In summary, the H19/miR-675-3p axis contributes to chemoresistance under hypoxia in CRC cells [17]. Thus, targeting H19 may overcome the chemotherapy resistance in various cancers.

### 7.2. Long Noncoding RNA EMS

The long noncoding RNA EMS forms a double-negative feedback loop with p53, promoting tumorigenesis by suppressing p53 translation [111]. Moreover, EMS links c-Myc to cell cycle regulation and tumorigenesis through E2F1 mRNA stability modulation [112]. Additionally, the lncRNA EMS is induced by hypoxia in esophageal cancer. Under hypoxic conditions, there is an increase in pre-mRNA-splicing regulator protein (WTAP) expression and a decrease in miR-758-3p levels. In esophageal cancer cell line ECA-109, the overexpression of both EMS and WTAP facilitates hypoxia-induced chemoresistance to cisplatin drugs, while elevated expression of miR-758-3p reverses this chemoresistance. Xenograft mouse model experiments proved that the knockdown of EMS/WTAP markedly attenuated the chemoresistance effect of cisplatin in EC cells [113]. Moreover, elevated levels of EMS and WTAP, along with reduced levels of miR-758-3p, are linked to poor survival outcomes in patients [113]. Thus, the lncRNA EMS/miR-758-3p/WTAP axis is crucial for regulating hypoxia-induced drug resistance to cisplatin [113].

### 7.3. Long Noncoding RNA ANRIL

Hypoxia also induced the expression of antisense long noncoding RNA in the INK4 locus (ANRIL) through the binding of HIF1-α to its promoter [114]. A dual luciferase assay showed direct interaction between miR-328 and its direct target, the drug resistance gene ABCG2 and ANRIL. ANRIL sponges miR-328, resulting in increased expression of ABCG2. Thus, the hypoxia-induced lncRNA ANRIL increases the resistance of retinoblastoma cells to cisplatin (DDP) by promoting cell proliferation, inhibiting apoptosis and boosting the expression of multidrug resistance proteins ABCG2 and MDR1 in Rb cells [114].

A study conducted on Midkine (MK), a hairpin-binding growth factor, indicated that MK promotes chemoresistance and carcinogenesis. ANRIL knockdown in tumor cells depicted reduced apoptosis and proliferation and increased levels of cisplatin sensitivity by impairing drug transporters MPRP1 and ABCC2. In the tumor microenvironment, stromal-derived MK from cancer-associated fibroblasts (CAFs) promotes cisplatin resistance by upregulating the lncRNA ANRIL, which induces the expression of multidrug-resistant proteins MRP1 and ABCC2 [115].

### 7.4. Long Noncoding RNA HOTAIR

Dysregulated expression of HOX anti-sense intergenic RNA (HOTAIR) is associated with different cancers like breast cancer [116], liver cancer [117] and lung cancer [118]. In breast cancer, it is associated with radiotherapy resistance due to recruitment of EZH2 to the MYC promoter [116]. HOTAIR over expression in invasive ductal carcinoma leads to increased expression of DNA repair factors like KU70, KU80, DNA-PK and ATM regulating breast cancer cell proliferation by regulating apoptosis and the cell cycle [116]. In NSCLC, HOTAIR induces gefitinib resistance through epigenetic regulation of EZH2 and H3K27me3. In PC9 lung cancer cells, overexpression of HOTAIR enhances H3K27me3 recruitment to p16 and p21 promoters. Silencing of HOTAIR induced the expression of p16 and p21, whereas CDK4, cyclinD, E2F1 and LSD1 expression declined [118]. Moreover, in glioblastoma (GBM), HOTAIR competitively binds to miR-526b-3p, limiting its ability to bind EVA1. Upregulation of EVA1 leads to temozolomide (TMZ) resistance in GBM [118].

In the human cervical cell line (HeLA), HOTAIR evades the inhibitory effect of Wnt/β-catenin pathway blocker ICRT14 due to its interaction with β-catenin [119]. In nasopharangeal carcinoma, HOTAIR contributes toward cisplatin resistance. It sponges miR-106a-5p and upregulates the expression of SRY box transcription factor 4 (SOX4), promoting NOC cell proliferation [120]. High expression of HOTAIR in colorectal cancer (CRC) is associated with a poor clinical prognosis. CRC cells with high HOTAIR expression display oxaliplatin resistance, whereas silencing of HOTAIR significantly increases its sensitivity. HOTAIR acts as a ceRNA for miR-1277-5p, resulting in upregulation of ZEB1. Thus, the HOTAIR/miR-1277-5p/ZEB1 axis modulates oxaliplatin resistance in CRC [121].

### 7.5. Long Noncoding RNA CBSLR

CBSmRNA-stabilizing lncRNA (CBSLR) is a hypoxia-induced lncRNA in gastric cancer cells. CBS can be oncogenic or act as a tumor suppressor, depending on the tumor type. It promotes colon cancer via autocrine and paracrine signaling, whereas its reduced expression promotes glioma tumorigenesis [122,123]. CBS has a distinct role in protecting different cancer cells like breast cancer, lung cancer, hepatocellular carcinoma and fibrosarcoma from ferroptosis [124,125]. Ferroptosis is an ROS-dependent form of cell death in which iron accumulation and lipid peroxidation are the main changes. Aberrant ferroptosis is associated with diverse pathological conditions and resistance to cancer therapies [126].

Overexpression of CBSLR is associated with poor prognoses and a worse chemotherapy response in gastric cancer (GC). HIF1-α induces lncRNA CBSLR to recruit YTHFD2 and CBS to form a CBSLR/YTHFD2/CBS complex, leading to decreased CBS mRNA stability via m6A modification [127]. Low expression of CBS favors the ubiquitin-mediated degradation of ACSL4 due to its reduced methylation. Silencing of CBSLR-upregulated ACSL4 at the post-transcriptional level increases the phosphotidyl ethanolamine level, leading to ferroptosis [127]. Thus, CBSLR/CBS/ACSL4 axis promotes ferroptosis resistance in the GC cells under hypoxia [127].

### 7.6. Long Noncoding RNA NORAD

Noncoding RNA activated by DNA damage (NORAD) is a highly conserved and abundant long noncoding RNA which plays a crucial role in maintaining genomic stability [128]. NORAD is upregulated upon DNA damage and has been implicated in various cancer-related processes, including cell proliferation, invasion, and metastasis [129]. Upregulation of NORAD is associated with tumor metastasis and poor patient prognoses in colorectal cancer (CRC) cells. NORAD is positively associated with HIF1-α. 5-Fluorouracil (5-FU) is the standard chemotherapy treatment for CRC. Upon hypoxia, the upregulation of NORAD leads to 5-FU resistance. NORAD acts as competing endogenous RNA (ceRNA) by sponging miR-495-3p to regulate HIF1-α expression [130]. The knockdown of NORAD inhibited the hypoxia-induced VM and 5FU resistance of CRC cells. Moreover, elevated NORAD expression has also been reported in cisplatin-resistant NSCLC cells. NORAD sponges miR-129-1-3p and boosts SOX4 expression, whereas its inhibition decreases cisplatin resistance in NSCLC cells [131]. Thus, targeting NORAD may overcome chemotherapy resistance in CRC and NSCLC.

### 7.7. Long Noncoding RNA NLUCAT1

A combined profiling study was conducted on early-stage lung adenocarcinoma (LUAD) and A549 cell lines cultured in hypoxic and normoxic conditions. The study suggested that nuclear lung cancer-associated transcript 1 (NLUCAT1), a particularly new nuclear hypoxia-regulated lncRNA transcript, was strongly upregulated by hypoxia in vitro and correlated with poor prognoses and other hypoxia markers in LUAD. A CRISPR-Cas9-mediated inactivation of NLUCAT1 showed decreased invasive and proliferative properties, an increased state of oxidative stress and high sensitivity to cisplatin-induced apoptosis. Transcriptome analysis of NLUCAT1 knockdown cells showed repressed genes present within antioxidant and cisplatin response networks. Partial mimics of NLUCAT1 inactivation in LUAD cells and increased ROS-dependent caspase activation were observed in cells with consequent knockdown of four particular gene products: PDK4, GLRX, GPX2 and ALDH3A1. In short, NLUCAT1 promotes aggressive phenotyping in early-stage hypoxic tumors [132].

### 7.8. Long Noncoding RNA LUCAT1

Another study demonstrated the underlying drug resistance mechanism of lung cancer-associated transcript 1 (LUCAT1), which facilitates the growth of CRC cells both in vivo and in vitro. In CRC cells, mechanistically, LUCAT1 interacts with the polypyrimidine tract-binding protein 1 (PTBP1), which results in the association of genes responsible for DNA damage with PTBP1. This phenomenon results in alternative splicing of DNA damage-related genes. A combination of LUCAT1 knockdown through antisense oligonucleotide (ASO) and chemotherapeutic drug delivery showed better outcomes in vivo when compared with a group treated with only chemotherapeutic drugs. Thus, CRC patients with higher LUCAT1 expression have correlated with poor prognoses and resistance to chemotherapy drugs clinically [132].

In glioblastoma stem-like cells (GSCs), LUCAT1 was found to be induced by nuclear factor erythroid 2-related factor 2 (NRF2) and hypoxia-inducible factor 1- α (HIF1α). LUCAT1 was found to be highly expressed in the hypoxic regions of glioblastoma (GBM). HIF1α and its co-activator CBP form a complex with LUCAT1 and regulate the genetic expression of the HIF1α target and GSC’s adaptation to hypoxia. In GBM xenograft models, silencing of LUCAT1 decreases tumor growth and promotes a longer survival rate in mouse models [133].

## 8. Challenges Associated with Targeting lncRNAs

Targeting hypoxia-induced lncRNAs for potential therapy in clinical settings could prove challenging due to their involvement in complex regulatory mechanisms. There is a significant risk associated with the involvement of off-target effects which can potentially disrupt the normal cellular function mechanisms [134]. This complexity arises because lncRNAs can interact with surrounding RNA molecules and proteins, which can cause unintended results in targeted therapies [134].

To address this challenge clinically, various combination therapies are being used which combine lncRNA inhibitors with traditionally available chemotherapy drugs to enhance treatment response and overcome chemoresistance [135]. Stimulus-responsive targeted nanoparticle delivery systems are also being devised which increase the specificity of delivering drugs to specific tissues. Hypoxia-induced lncRNAs can serve as a better source of such targeted therapies [136].

## 9. Conclusions

Hypoxia contributes to resistance to therapy through multiple mechanisms, such as inhibiting apoptosis, regulating autophagy, responding to DNA damage and facilitating drug efflux. The expression of lncRNAs specific to different tissues and their role in distinct resistance mechanisms to various drug types highlight their potential as therapeutic targets. This review highlighted the intricate relationships among lncRNAs, hypoxia and their target genes in the mechanisms behind chemoresistance. Gaining insight into these interactions is essential for creating new therapeutic approaches to combat drug resistance in cancer therapy. By targeting lncRNAs, it may be possible to overcome drug resistance in cancer patients, indicating their promise as effective therapeutic targets and enhancers of chemotherapy.

## 10. Future Directions

Further research on deciphering the role of lncRNA-related drug resistance could lead to new strategies for enhancing the outcomes of cancer treatment.
